# A bioengineering perspective on modelling the intestinal epithelial physiology in vitro

**DOI:** 10.1038/s41467-020-20052-z

**Published:** 2020-12-07

**Authors:** Maria Antfolk, Kim B. Jensen

**Affiliations:** 1 BRIC – Biotech Research and Innovation Centre, Faculty of Health and Medical Sciences, University of Copenhagen, Copenhagen, Denmark; 2 Novo Nordisk Foundation Center for Stem Cell Biology (DanStem), Faculty of Health and Medical Sciences, University of Copenhagen, Copenhagen, Denmark; 3 Department of Biomedical Engineering, Lund University, Lund, Sweden

**Keywords:** Gastrointestinal models, Intestinal stem cells, Gastroenterology, Tissues

## Abstract

The small intestine is a specialised organ, essential for nutrient digestion and absorption. It is lined with a complex epithelial cell layer. Intestinal epithelial cells can be cultured in three-dimensional (3D) scaffolds as self-organising entities with distinct domains containing stem cells and differentiated cells. Recent developments in bioengineering provide new possibilities for directing the organisation of cells in vitro. In this Perspective, focusing on the small intestine, we discuss how studies at the interface between bioengineering and intestinal biology provide new insights into organ function. Specifically, we focus on engineered biomaterials, complex 3D structures resembling the intestinal architecture, and micro-physiological systems.

## Abstract (web summary)

Maria Antfolk and Kim Jensen discuss how to model intestinal epithelial cell function in the dish and how various physiologically important environmental conditions, for example, extracellular matrix, pressure and flow, can be modelled and how this is applicable to clinical work.

## Introduction

**Fig. 1:**
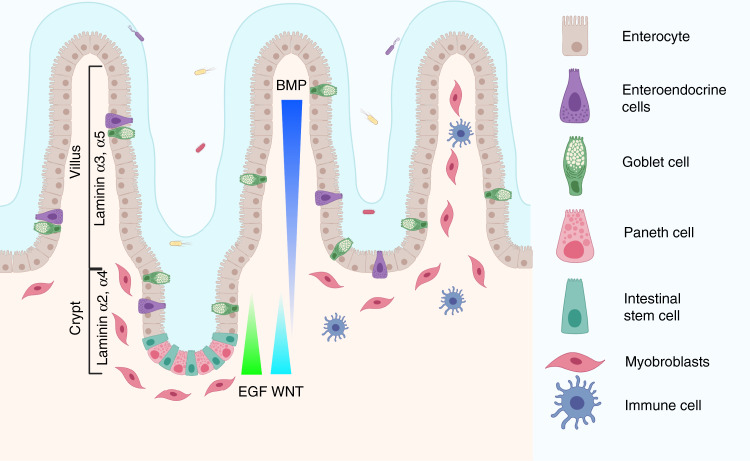
Structure of the small intestine. The intestinal topography includes villi, where the absorptive enterocytes, secretory enteroendocrine and goblet cells reside, and crypt domains harbouring the intestinal stem cell niche including stem cells and Paneth cells. Underlying mesenchymal cells like myofibroblasts support the epithelium by secreting important growth factors such as WNTs, R-spondins and BMP antagonists. Gradients are believed to exist for the factors promoting stem cell self-renewal, including WNT and ligands for the ErbB pathway, whereas opposing gradients exist for differentiation-promoting factors such as BMP. The gradient, indicated here for EGF, represents all the ligands of the family, including Nrg1, Areg, Hbegf and Ereg^107–109^, which support intestinal stem cell function. Many immune cells also reside in the intestine, constantly monitoring microbe and food antigens. Created with BioRender.com.

In the small intestine, the process of digestion occurs in symbiosis with billions of microbes residing in the lumen of the intestine. Here, the epithelial cell layer that faces the lumen forms a protective barrier that shields the body against direct exposure to microbes and food antigens. This layer is organised into finger-like protrusions (villi), which serve as the site of nutrient absorption and pockets (crypts) containing mostly proliferating cells. This outer layer of intestinal epithelial cells is supported by the underlying stroma containing mesenchymal cells, neurons and vasculature^1^. Throughout life, the epithelium is continuously replenished by intestinal epithelial stem cells that reside at the bottom of crypts. These stem cells are highly proliferative and will constantly give rise to progeny that move up along the crypt–villus axis, first transitioning into transit amplifying (TA) compartment, before they end up as differentiated post-mitotic cells on villi and eventually being shed into the lumen. These differentiated cells actively participate either in the absorption of nutrients (enterocytes) or in the conditioning of the environment as secretory cell types. Some of these cells, such as goblet, enteroendocrine and tuft cells, are primarily located on villi, while Paneth cells are found at the crypt bottom, where they are intercalated between stem cells and contribute to the intestinal stem cell niche (Fig. 1)^2,3^. It has, however, been very difficult to study the impact of tissue architecture, host–microbe interactions and general tissue replenishment in a refined manner using animal models or traditional cell culture techniques.

**Fig. 2:**
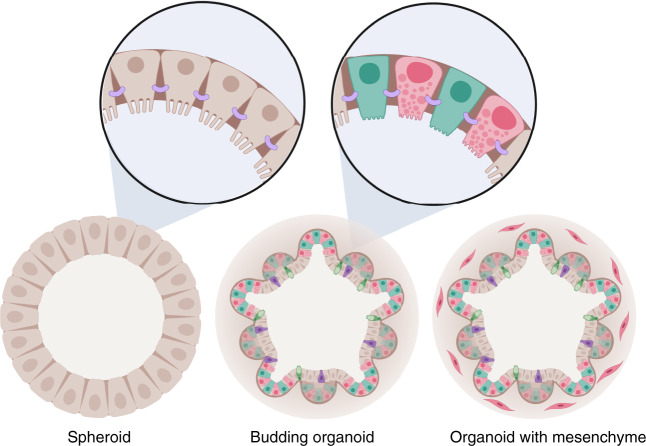
Different types of organoids. Intestinal organoid architecture comes in different flavours ranging from spherical structures to budding organoids (brown enterocytes, turquois intestinal stem cells, pink Paneth cells, purple enteroendocrine cells and green goblet cells) with distinct localisation of different cellular domains containing stem cells and differentiated cells, and organoids that include a supporting mesenchyme (illustrated with pink cells surrounding the organoid). The cells are arranged with the apical membrane inwards and with appropriate cellular junctions that normally support the formation of an intact barrier including tight junctions (purple). Created with BioRender.com.

Using classical cell culture methods primary intestinal epithelial cells could not be kept in culture for longer than a few days. Cancer cell lines cultured on plastic or on porous membranes have consequently been used for studying signalling in the intestinal epithelium^4^. Although these lines have provided us with instrumental knowledge, they have obvious limitations in addressing some of the unresolved questions in intestinal epithelial biology, such as how the natural heterogeneity is maintained in the intestine, which signalling mechanisms control cell fate decisions and how stem cells are maintained in an undifferentiated state for extended periods of time. This all changed with the advent of two complementary methods developed by the groups of Hans Clevers and Calvin Kuo. They reported that primary intestinal epithelial cells could be cultured long-term in three-dimensional (3D) scaffolds either as self-organising epithelial organoid units^5^ or as mixed cultures of epithelial and mesenchymal cells^6^. Human- and mouse-derived intestinal organoids can now be generated from single stem cells or crypts isolated from primary fetal or adult tissues^5,7^, embryonic stem cells or induced pluripotent stem cells (iPSCs)^8^ and can be propagated long-term as spherical or budding organoids^5,9,10^ (Box 1 and Fig. 2). As such these organoid models comprise near-physiological systems that provide the opportunity to complement studies using animal models, and address questions related to early human development of the intestine using stem cells and differentiated epithelial cells derived from a number of different sources (Table 1).

**Table 1:** Characteristics of different organoid models for the small intestine derived either from tissues or from pluripotent stem cells.

Cell source	Species	Culture characteristics	Matrix	Supplementary requirements	Special feature	Disadvantages	Advantages	Reference
Adult small intestine	Mouse	Budding and spherical	Collagen	R-spondin, EGF, Noggin	Cultures at the air–liquid interface	No evidence of passaging, reported for early neonatal tissue source	Easier to culture, and source	^6^
	Mouse	Budding	Matrigel	R-spondin, EGF, Noggin			Can be cultured from all stages	^5^
	Mouse	Spherical	Collagen type I	R-spondin, EGF, Noggin, WNT	Induces a wound healing phenotype			^53,61^
	Human	Spherical	Matrigel	WNT, R-spondin, EGF, Noggin		More challenging to culture, and source		^9^
	Human	Budding	Matrigel	WNT, R-spondin, EGF, Noggin, IGF-1, FGF-2		More challenging to culture, and source	Closest to human in vivo	^44^
Fetal small intestine	Mouse	Spherical	Matrigel	(R-spondin), EGF, Noggin		Does not mature spontaneously	Easier to source than human fetal tissue	^7,113^
	Human	Spherical	Matrigel	WNT, R-spondin, EGF, Noggin, PGE2		More challenging to culture, and source		^7^
hPSC-derived	Human	Spherical, with mesenchyme		EGF, Noggin, R-spondin1	More similar to human fetal primary cells	Time consuming to establish, expert knowledge in PSC required	Easier to source than human primary tissue	^8^
	Human	Spherical, without mesenchyme	Matrigel	EGF, Noggin, R-spondin, CHIR9902	More similar to human fetal primary cells	Time consuming to establish, expert knowledge in PSC required	Pure epithelial PSC-derived population	^114^

As a primary cell source, with a genome reflecting either healthy individuals or patients, and with multilineage differentiation potential in vitro, the organoid system is attractive for disease modelling. Furthermore, elegant studies have demonstrated that organoids can be genetically engineered in vitro to study the effect of individual genes^11,12^ or screen for genes with important specific functions^13–15^. In this way, organoids from patients have been used to model diseases such as cystic fibrosis^16^, inflammatory bowel disease^17–19^, enteropathies including congenital diarrhoeal disorders^20^, and for modelling host–pathogen interactions including infection with *Cryptosporidium*^21^, *Salmonella*^22,23^, *Clostridium difficile*^24^, and entero-^35^, noro-^36^ or coronaviruses^25,26^. Thus, the development of the organoid technology has already had great impact on our ability to study functional aspects of the physiology of intestinal epithelial cells from a range of different species including humans. Yet, we have to keep in mind that these approaches are reductionistic given that only one component of a complex tissue is studied in isolation and often in ECM derived from tumour tissues. It is consequently important to stress that there is significant room for improving the methodology, before it reaches a state where we should consider this as a replacement for animal models. Future studies are likely to guide us in this direction with more refined cellular models using defined components or enhanced structural guidance of the epithelium.

Box 1 Definition of the organoid Historically, the word *organoid* has had different meanings. During the 1950s and 1960s the word was used predominately to describe intracellular organelles^110^, and used to describe tumour formation^111^. In a third case the word was used to describe spontaneous formation of organ-like structures from cultures of embryonic tissues^112^, closer to today’s use of the term organoid.

The term *organoid* was reintroduced in 2009 to describe the structures that were formed when epithelial cells from the small intestine were cultured in Matrigel in the presence of epidermal growth factor (EGF), Noggin and R-spondin1^5^. The term has subsequently been applied to cultured 3D structures from other organs including colon, pancreas, mammary gland, brain, retinal epithelium, cerebellum, stomach, lung, salivary gland, thyroid, liver and kidney^10^. Only a small subset of these tissue organoids recapitulates the in vivo morphology to the same extent as the mouse small intestine, where organoids form distinct crypt and villus domains.

The definition of an organoid upon the reintroduction of the term in 2009 has since not reached a full consensus. It has been implied that organoids should develop from stem or progenitor cells, be constituted by multiple organ-specific cell types, self-organise into a 3D structure and recapitulate some organ-specific functions.

Here, we have chosen a somewhat broader definition to include all different ways in which the word organoid is currently used. In this review we define *an organoid as a self-organising 3D structure, grown and expanded* in vitro*, and consisting of organ-specific cells with a restricted lineage commitment and having organ-like functions*. Our definition, therefore, also encompasses spheroids (or enteroids, as is a term used for spheroids derived from the small intestine and colon), where symmetry breaking events are less apparent.

Our definition avoids restricting the origin of the organoid to stem cells and allows the inclusion of tumour organoids, where the debate about the existence of the cancer stem cell is not yet settled.

## Modelling the intestinal epithelial physiology in vitro

### The intestinal stem cell niche

The intestinal stem cell niche represents a unique biophysical and biochemical microenvironment that supports stem cell self-renewal and maintains the epithelial cells in an undifferentiated yet proliferative state^27–29^. The conductive environment in the niche is provided by complex interactions with neighbouring epithelial cells including Paneth cells, different mesenchymal cell populations including the underlying muscle, enteric neurons, immune cells as well as the endothelium^2,30–33^. Within this environment stem cell behaviour is controlled by the local production of signalling molecules including Wingless-type MMTV integration site family members (WNTs), bone morphogenetic protein (BMP) inhibitors and members of the epidermal growth factors family^34,35^. In combination with an extracellular matrix (ECM), this bare minimum is sufficient to support stem cell cultures in vitro^5^. Interesting new observations also support that the gut microbiome via break-down of food into, e.g. short chain fatty acids, play a role in controlling stem cell behaviour and energy metabolism^36–38^ and that crypt structure, at the same time, provides a protective environment for exposure to other harmful microbial metabolites^39^.

An additional essential component of the intestinal stem cell niche is the ECM that separates the epithelium from the underlying stroma, and which provides a structural component of the niche that influences cell fate choices. Careful characterisations of ECM components have revealed that although some components like collagen IV and fibronectin are present ubiquitously along the crypt–villus axis others show distinct expression patterns^40,41^. Laminins, which are a major constituent of the ECM, are composed of three subunits (α, β and γ) that via their α unit, form ligands for integrin cell surface receptors. Interestingly, laminins containing α2 and α4 subunits are detected at the bottom of crypts, whereas the α3 and α5 subunits are associated with the differentiated villus compartment and they could consequently have a functional role in controlling cell behaviour^40–42^. The exact roles of the different ECM components in the basement membrane are currently not known. In order to shed light on the role of individual ECM components, it will be advantageous to utilise in vitro systems where cells can be spatially organised in a controlled microenvironment.

### The basic requirements for intestinal epithelial cells

The methods for growing organoids revealed that the recurrent mutations in colorectal cancer (loss of APC (WNT activation), activating mutations in Ras (growth factor signalling), and loss of SMAD4 (BMP signalling)) in combination with an ECM provide the minimum signalling requirements for stem cell maintenance of intestinal epithelial cells^5,9,43^. Here, R-spondin supports stem cell self-renewal as an agonist of the WNT pathway, epidermal growth factor (EGF) family members stimulate proliferation via growth factor signalling, and Noggin suppresses differentiation as an antagonist of bone morphogenetic protein (BMP)-induced differentiation^5^. Importantly, these minimal culture conditions (EGF, Noggin and R-spondin1 (ENR)) support cultures of epithelial cells derived from the mouse small intestine, where secretion of WNTs from Paneth cells is sufficient to support stem cell self-renewal^2,5^. For cultures of human small intestinal epithelial cells an exogenous source of WNT has to be provided^9^. These conditions provide the core signalling requirements for expansion of intestinal epithelial cells. Interestingly, recent work demonstrates that addition of Insulin-like Growth Factor 1 and Fibroblast Growth Factor 2 to this core growth factor cocktail enhances both clonal growth and lineage potential^44^. With the advances in the culture conditions the physiological relevance of the organoid systems is enhanced, yet, the influence of biophysical cues on lineage choices remains largely unexplored. This is what we will discuss in the following.

### Two-dimensional open monolayer cultures

Even though organoid cultures have proven an extremely versatile tool, there is an interest in using primary intestinal epithelial cells in monolayer cultures for multiple reasons.

Firstly, the small intestinal epithelium in vivo is constituted by a single cell layer supported by the submucosa. Here, the apical surface is exposed to the luminal content and the shear stresses from the movement of content along the length of the gastrointestinal tract. In organoids, the epithelium is polarised with the basolateral membrane facing outwards and the apical membrane facing towards the hollow lumen (Fig. 2). This complicates studies that involve natural barrier translocation or microbial–epithelial interaction studies, where the initial interaction occurs at the luminal cell surface. This caveat can be bypassed by microinjecting microbes or compounds into the organoid lumen, however, this is a cumbersome process and difficult to automate^23^. Two-dimensional culture systems are consequently tractable either as classical adherent cultures or placed on top of a supporting scaffold providing access to the apical membrane. Alternatively, this type of topology could also be achieved using organoids with an inversed morphology^45^.

Secondly, the majority of methodologies for imaging and manipulating epithelial cells have been developed for 2D cultures, although recent advances in light sheet microscopy are enhancing the imaging of 3D structures^46^. It has, however, proven challenging to establish 2D culture systems supporting both stem cell self-renewal and differentiation of epithelial cells. Most described monolayer cultures consequently constitute a means to an end, where cells seeded in 2D lose their ability to self-renew and eventually become terminally differentiated^47^. Despite these limitations, short-term monolayer cultures of intestinal epithelial cells have proven useful for assessing the function of growth factors/morphogens on the behaviour of intestinal epithelial cells including lineage choices^48^. Moreover, refined monolayer-culture methods provide strong indications that new medium compositions might facilitate long-term culturing of primary intestinal epithelial cells in 2D from both human and mouse^49–51^. Interestingly, recent studies assessing stem cell functions in 2D report that cells, even under these conditions, self-organise into proliferative regions expressing markers of stem cell and Paneth cells, and domains of differentiated enterocytes, goblet, tuft and enteroendocrine cells^48–51^. These are important observations, which for now provide testable models for assessing whether the mechanism that allow cells to self-organise is the same in 2D, 3D and in vivo, and also how topography might influence cell fate choices, patterns of differentiation and cellular maturation.

### The importance of the matrix for growth

ECM components represent an important element of the stem cell niche and are instrumental for providing the specific biophysical environment that controls cell fate. Using refined models such as organoids, it is now possible to modulate the properties of the ECM and assess how this influences cell fate choice. Such refined questions could not be addressed with cancer cell lines given that the natural mechanisms regulating cell fate choices are compromised. There are, however, a number of aspects that should be considered.

Firstly, it is necessary to characterise the cellular system used. The mesenchyme naturally represents a major source of ECM components. As an elegant example illustrating the guiding properties of the matrix produced by the mesenchyme, organoids that are co-derived with mesenchymal populations from PSCs can be cultured in gels completely lacking adhesive support^52^. It is consequently difficult to perform a refined study of how specific matrix components affects cell fate, if the epithelial cells are sheltered from the matrix by mesenchymal subpopulations producing their own ECM components.

Secondly, one needs to consider the composition of the matrix. Matrigel or basement-membrane extracts are the most widely used ECM to support the growth of intestinal epithelial organoids and are composed of laminin (Lam111 ~60%), type IV collagen (~30%) and nidogen (~8%), as well as a number of growth factors. Type I collagen has also been widely used as a matrix scaffold; however, here results have been somewhat diverging. Unlike Matrigel, type I collagen does not by itself support the growth of murine intestinal epithelial organoids in the presence of EGF, Noggin and R-spondin1, unless WNT is supplied as a purified component^53^. However, epithelial organoids can be maintained in type I collagen in conditions that include mesenchymal cells^6,54^. Yet, it remains to be shown whether the mesenchyme under these conditions is shielding the epithelium from the type I collagen via de novo matrix production, secreting high levels of WNT thereby bypassing the need for an exogenous source, or that the matrices used for the different studies differ in their biophysical properties. Apart from these two more widely used matrices, mixtures of fibronectin and laminin have been explored as a more defined matrix^55^, and decellularised small intestinal submucosa as a natural scaffold^56^. Despite the partial successes of these matrices, engineered protein gels provide clear advantages for addressing how the specific properties of the ECM support the intestinal epithelium.

Thirdly, bioengineering methods now allow the generation of artificial matrices based on individual components that confer specific properties. It is consequently possible to decouple biophysical properties from biochemical cues, so that one variable can be studied at a time. Using these methods, it is possible to fine-tune, e.g. the stiffness of the matrix, pore sizes and degree of cross linking of different components, and introduce various linker elements that can be cleaved by the cells in the organoids upon secretion of, e.g. matrix metalloproteinases (MMP)^57–60^.

### Bioengineering new stem cell niches

Much of the research performed in vitro has until now relied on the self-organisation of cellular structures using either cell lines or primary cells to recapitulate the intestinal epithelium. The development of advanced bioengineering approaches provides new and exciting possibilities for directing the organisation of cells in controlled environments. The tools facilitate engineering 3D matrices and materials with defined sizes, structures, porosities, stiffnesses and biochemical profiles, and can be complemented with dynamic features such as controlled shear stress, mechanical stimulation and motion. By controlling the microenvironment through bioengineering approaches, it is consequently possible to obtain greater insights into the mechanisms that ultimately control cell fate.

### Engineered 3D matrices

Tumour‐derived basement-membrane extracts are the most widely used matrix for cultures of intestinal epithelial organoids^5^. The composition of these matrices is, however, poorly defined and the production is associated with batch-to-batch variability, making it difficult to completely standardise studies that focus on the specific interactions between the microenvironment and the cells. The issues of variation between batches and the fact that it is extracted from animal tissue also complicate the direct translation of current organoid technologies into regenerative therapies^1^. Collagen gels have been proposed as an alternative to these poorly defined matrices^61^. It is, however, clear that the specific properties of the matrix will have significant impact on cell fate decisions, and the matrix used therefore needs to be tailored to specific needs and that it is difficult to extrapolate findings from one matrix to the other^53^.

**Fig. 3:**
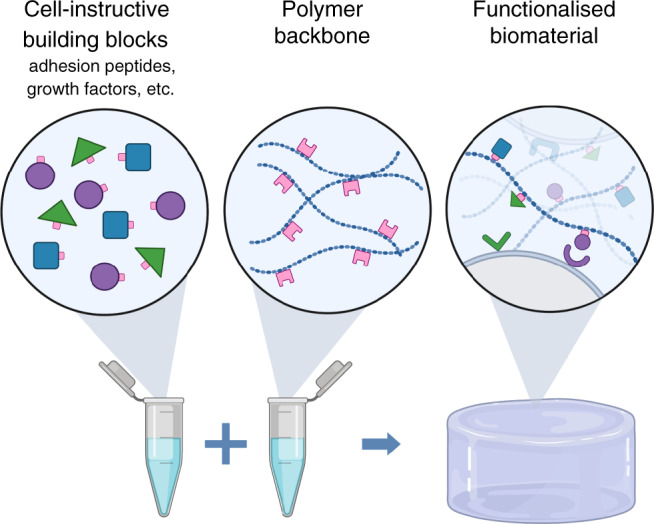
Synthetic matrices for stem cell research. Synthetic matrix assembly using, e.g. click-chemistry, resembling the use of toy building blocks where cell-instructive building blocks of choice, e.g. adhesion peptides or growth factors, are combined with a polymer backbone to form a functionalised scaffold where cells can adhere and be cultured. Created with BioRender.com.

As a complement to natural matrices, artificial materials have been utilised for supporting the growth of intestinal organoids^62,63^. These synthetic matrices provide a new and refined context for assessing how cell fate choices are influenced by extracellular components and thereby the environment. Their synthetic nature offers parallels to toy building blocks where different pieces can be combined independently and in a patterned manner, using click-chemistry, thereby allowing control of both the biochemical environment, with adhesion opportunities and growth factor densities, and the biophysical properties including stiffness and porosity (Fig. 3).

Cell adhesion to the biomaterial, proliferation and differentiation can be stimulated by incorporating adhesion ligands such as the Arg-Gly-Asp (RGD) peptide or full-length proteins such as laminins. By varying the cross-linker concentration the biophysical properties including the material stiffness and nano-porosity can be controlled^64^. Importantly, ligand display and material stiffness can be varied independently in synthetic biomaterials, allowing for a greater control of the experimental parameters. The biocompatibility of the material can also be improved by incorporating elements improving biodegradability through MMP-sensitive cross linkers^57^. Within the 3D space of a biomaterial such elements promote cell spreading, which is especially important for larger cellular structures such as organoids to be able to form and grow^63^. In addition, microporosity can be introduced during the scaffold formation, allowing the cells to instantly spread through the material without having to degrade the biomaterial first^65^. To dynamically modulate the stiffness of the biomaterial and introduce a gradual softening, e.g. acrylate groups can be introduced, where the ester bonds of this group will undergo hydrolysis and gradually soften the biomaterial over a course of days^62^. Instead, by introducing allyl sulphide groups, the biomaterial is rendered photodegradable, which allows for instant softening or even full degradation in a matter of seconds when exposing the biomaterial to UV light^66^. Using this method local spatial softening of the biomaterial can also be introduced, directing the budding or crypt formation of an intestinal organoid.

Using synthetic matrices Gjorevski et al. elegantly illustrated how these matrices can be applied to address biophysical requirements for mouse organoidsʼ formation starting from single intestinal stem cells. Studying the process from formation and growth into fully mature structures, the authors assessed how this is dynamically supported by the environment. The authors revealed that this is a multistep process with phases that have discrete requirements^62^. By adding a dynamic aspect to a semisynthetic polyethylene glycol (PEG)-based matrix, including the RGD-peptide and laminin-111 for enhanced cell adhesion, they provided a degradable matrix that initially had the optimal stiffness for organoid formation (*G* = 1.3 kPa) and in the second growth phase became softer allowing organoids to expand as budding structures (*G* = 190 Pa)^62^. The healthy small intestine has been measured to have Young’s modulus of *E* = 2.9 kPa^67^. With the relationship in biological tissues being *E* = 3*G*^68^, this would correspond to *G* = 967 Pa, indeed in the range observed to be optimal for culturing intestinal epithelial organoids. Interestingly, a subsequent study found that a PEG-based matrix functionalised with the cell adhesion domains from type I collagen (GFOGER) on its own was sufficient as a matrix in supporting the growth of primary human intestinal epithelial cells as spheroids^69^. This again points to the important role of biomechanical cues in controlling cell fate and is supported by the observation that organoid ‘crypt’ formation depends on local mechanical signals as the length and the number of crypts per organoid can be modulated by softening the matrix^70^. A complementing study using high-resolution live-imaging reported that organoid formation in basement-membrane extracts go through two distinct phases similar to organoids grown in synthetic matrices. Here, the first phase is characterised by expansion as a spheroid, followed by budding and differentiation. Mechanistically, the transition between the two states is, both in the synthetic matrix and the basement-membrane extract, driven by a reduction in the mechanosensory YAP signalling pathway^46,62^.

Synthetic matrices have also been applied to hPSC-derived intestinal organoids illustrating that these can also be grown in a PEG-based matrix. Here, the matrices functionalised with the RGD peptide, the cell adhesion domains from type I collagen (GFOGER) or laminin α1 subunit (IKVAV or AG73) all supported survival of the organoids, although the scaffold containing the RGD peptide had the greatest functionality. The synthetic matrix also contained a protease-cleavable peptide to allow for modulation of the matrix, but the importance of this modality was not reported^71^. Notably, the requirements for mouse intestinal epithelial organoids were surprisingly more complex than those described for hPSC-derived organoids. However, here it is important to keep in mind that the hPSC-derived organoids used in these studies also contain mesenchymal cells. This will consequently provide an additional source of ECM components. It is therefore difficult to perform direct comparisons between the two systems. In fact, it has been shown that these hPSC-derived organoids can be cultured in matrices completely lacking adhesive support, illustrating that one important function of the associated mesenchyme is to provide a functional extracellular scaffold^52^. It will be exciting to follow how bioengineering can be exploited to reveal the impact of the biochemical and biophysical environment on intestinal stem cell behaviour. Here, the PEG-based matrix without adhesive properties could provide instrumental insight into deciphering how the supporting mesenchyme naturally supports epithelial stem cell properties. However, to pinpoint the requirements for the epithelial cells, it is essential to work in much more reductionist models such as pure epithelial cultures from either mouse or human tissues.

### Complex 3D strategies for structural guidance

**Fig. 4:**
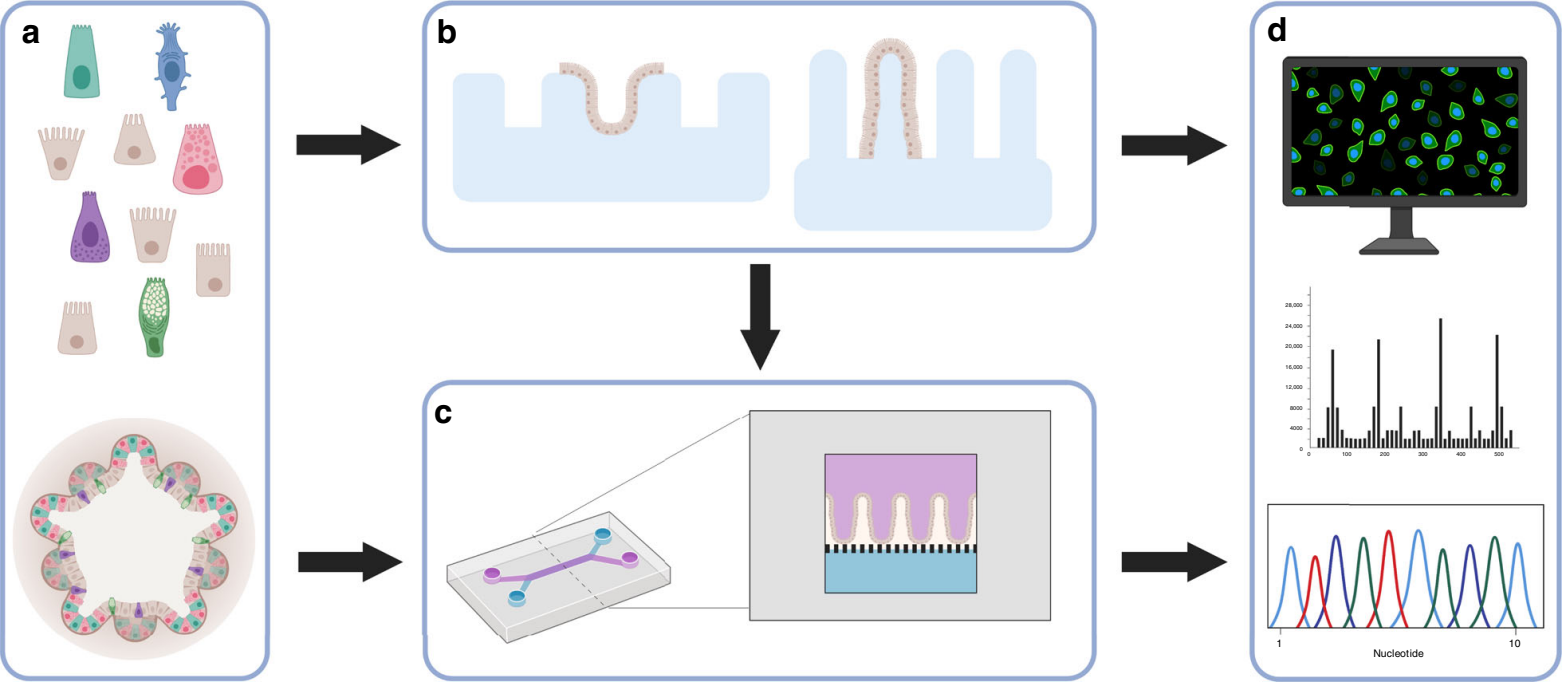
Establishing complex 3D structures for organ mimicry and dynamic micro-physiological systems. **a** Either single cells or organoid fragments can be used to seed these systems. **b** Complex 3D structures, e.g. crypt-like or villus-like, have been used to guide the cellular organisation. These structures can be fabricated from, e.g. biomaterials, PDMS, silicon or plastics such as polystyrene. **c** Intestine-on-a-chip devices have been used to create dynamic culture conditions including shear stress from fluid flow as well as cyclic mechanical deformation that resemble the natural movements associated with peristalsis. Intestine-on-a-chip device seen from above with an upper (purple) and a lower (blue) channel. Cross section of the device which is commonly composed of an upper (purple) and a lower (blue) channel separated by a porous membrane. Epithelial cells can be seeded into the upper channel, e.g. as a flat 2D layer or on a 3D construct that provides topographic features such as villi (as shown in the figure). These devices are most often made of PDMS, a transparent polymeric organosilicon, also making them ideal for live-cell imaging. **d** The intestinal barrier can be assessed by introducing various compounds to the upper surface of the 3D constructs, or through the inlet of the intestine-on-a-chip with access to the apical side of the epithelium. Medium can subsequently be collected from below the 3D construct or through both channel outlets of the intestine-on-a-chip for further analysis. In addition, cells seeded on these structures can later be isolated for, e.g. gene or protein expression analysis. Moreover, cells can be directly monitored to follow cell behaviour, using live-cell imaging. Created with BioRender.com.

Although organoids have proven a versatile tool for addressing the key questions in intestinal epithelial biology, their form is not optimal for studies focusing on barrier function and interactions with the luminal surface of the epithelium. Moreover, topographic constraints that will exert divergent mechanical forces on the tissues as well as provide a basis for growth factor gradients are difficult to recapitulate when cells are cultured in a 3D matrix. A range of 2D topologies have consequently been investigated to overcome not only the closed nature of the organoid structures but also to assess how additional architectural features such as tube structures and scaffolds shaped with villi and crypts affect cell behaviour^72–75^ (Fig. 4 and Box 2). Although the application of structural guidance based on scaffolds mimicking the stromal surface of the intestine is still in its infancy, such technologies might provide a framework for advanced studies of complex interactions between microbes and the intestinal surface^76^. Here an important study recently revealed that an intestinal epithelium can be maintained long-term in hydrogel-based microchannels formed to include crypts and support laminar flow through the device. In this patterned structure, stem cells were located at the bottom of the crypts, whereas differentiated cells were facing the lumen facilitating studies of regeneration and host–microbe interactions^77^. It will be central for the field to follow how this technology further develops.

From a physiological perspective the architecture of villi is extremely important, as they increase the surface area of the small intestine and thereby the nutrient absorptive capacity by more than 30-fold. Villi are finger-like protrusions extending 0.5–1.6 mm into the lumen of the small intestine, and are composed of an epithelial surface on top of an underlying stroma that includes myofibroblasts, blood vessels and lymphatics. Studies of the fetal intestine suggest that the topology of the adult small intestine does have a function. In contrast to adult villi that are covered by post-mitotic and terminally differentiated epithelial cells, the smaller rudimentary villi found in the fetus are linked with an immature phenotype of the epithelial component. Importantly, the transition into the mature adult phenotypes coincides with emergence of the crypt–villus axis^78^. It remains an open question how the villi could play an active role in the cellular organisation and directing cell fate choices within the epithelium.

There could be multiple explanations for why the structure would guide cell fate choice either directly or indirectly. At one level it is worth considering cell shapes. As cells translocate from crypt bottom to the villus top, there is a gradual decrease in the fraction of the cell membrane that faces the ECM, when compared to the apical counterpart. At the hinge that forms, where cells exit the crypt and enter the villus, this aspect changes dramatically^79^, and such changes could have significant consequences on mechanosensory pathways and thereby cell fate decisions. In addition to form, it is clear that the crypt–villus axis in vivo serves an important function in establishing gradients of factors that are maintaining stem cell self-renewal, e.g. WNTs and pro-differentiation signals such as BMP. This supports the spatial confinement of stem cells and differentiated cells, and elegant genetic studies have demonstrated that interfering with these gradients is sufficient to interfere with the cellular boundaries^80,81^. Similarly, flow across a cell membrane can act not only via shear stress on the cell surface but also by removing or transferring substances from cells nearby. Structural guidance via bioengineered solutions represent elegant tools for specifically addressing the importance of these individual components and mechanisms.

In line with the spatial confinement of differentiated and non-differentiated cells in vivo along the crypt–villus axis, studies of Caco-2 cells on various scaffolds, mimicking the morphology of the small intestine with crypts and villi, indicate that 3D architecture affects the expression and organisation of cells into proliferating and differentiating domains^82,83^. Interestingly, a complementing study using primary human intestinal epithelial cells cultured on a similar 3D scaffold did not reveal any type of confinement of cells with a particular phenotype. Instead, proliferating cells were found along the entire crypt–villus axis. Mimicking the natural system, growth factor gradients had to be applied along the crypt–villus axis to confine undifferentiated cells in the crypt domain and differentiated cells on the villi. Here, the authors supplemented the medium under the device with WNT3a, R-spondin3 and Noggin, and the medium above the device with the Notch-inhibitor (DAPT), which is known to induce secretory differentiation. Using a nano-porous biomaterial allowed for the formation of stable linear gradients across the cellularised device^84^. The 3D architecture, as observed during fetal intestine development, is consequently not in itself sufficient to impose strict cell fate decision, but can provide the basis for changes in growth factor availability and potentially also the probability to respond to specific cellular signals. The future will reveal how the implementation of new technologies from the bioengineering field such as 3D printing of different matrix components as well as different cell types will change studies of the intestinal epithelium. Here, one area of interest will be to address how topography instructs cells via structure and also how growth factor gradients are first established within the tissues.

Box 2 Microfabrication of biomimetic small intestinal models Complex micropatterned 3D structures resembling the small intestinal architecture are commonly fabricated using microfabrication processes and micromoulding. A master mould for the biomaterial scaffold can be fabricated by utilising either photo lithography^84^, micromilling, laser ablation^115^ or 3D printing. Using these master moulds, a more flexible mould of PDMS is usually casted^84,115^. Sometimes another moulding step is also utilised to improve the detachability^115^ of the biomaterial scaffold or work as a sacrificial layer^74^ to facilitate the fabrication of softer or more brittle scaffolds. An exciting new opportunity for sculpting scaffolds as an alternative to micromoulding is 3D bioprinting and it will be interesting to follow how this technology will impact the bioengineering arena^116^.

Irrespective of the method, the scaffolds need to possess a high enough stiffness to not break during the fabrication process or deform under its own weight or due to cellular traction forces. Consequently, these scaffolds are limited in the stiffness that can be used. As previous work on organoids showed that softer scaffolds are required for more complex growth patterns when working with primary cells^62^, it will be interesting to further monitor the development of additional biomaterials that support not only spheroid growth but also budding into functional domains of organoids derived from different cell sources. It does, however, still remain unclear whether the observations related to stiffness and cell fate are directly translatable from organoid studies, but future studies will hopefully help elucidate this.

### Organ-on-a-chip

The emerging organ-on-a-chip technology has enabled new and innovative ways to study organ level functions, and provides an alternative technology for complementing studies on health, tissue development and disease^85^. The technology utilises microfluidics and microtechnology in combination with cell culture models to recapitulate tissue- and organ-level physiology in a dynamic mode that has been difficult to emulate using classical static cell culture methods. The organ-on-a-chip device represents a minimal functional unit, such as a barrier or tissue interface, required to model selected in vivo functions of the organ. The device is built using electronic microchip manufacturing methods, whereby micro-sized cell culture chambers can be fabricated with high precision. This allows for media perfusion and mechanical stimulation. These systems recapitulate aspects of the native tissue to a higher degree than conventional 2D or 3D culture systems, and have the capacity to model tissue–tissue interfaces, shear stresses induced by fluid flow, tension and compression, which are part of the mechanical motion of tissues. Importantly, despite the name, these devices are not meant to recapitulate whole organ physiology.

In a conventional macrofluidic system such as a traditional bioreactor the fluid regime is characterised by a turbulent flow, inducing mixing, whereas the flow in a microfluidic system is laminar. The microfluidic system consequently offers greater control over the fluid flow, and it is easier to control the impact on cells within the device. Furthermore, microfluidic devices provide the opportunity for high-resolution on-line monitoring, via measurements leading to minimal sample and reagents consumption and real-time imaging^86,87^. Importantly, these systems also offer the opportunity to simulate relevant interactions between different cell types. One example is the addition of immune cells to tubes covered with endothelial or epithelial cells and the ability to remove these cells from the system through flow, thereby emulating the body’s blood or lymph system.

Organ-on-a-chip devices are generally made up of an upper and a lower microfluidic channels separated by an ECM-coated porous membrane on which cells are seeded creating a surface, e.g. for studies of barrier function^88^ (Fig. 4). Polydimethylsiloxane (PDMS) is commonly used as a fabrication material for these devices. This silicone elastomer has a limited ability to support cell adhesion, but can be modified with ECM proteins to allow cells to adhere^89^. The first studies using these principles in combination with Caco-2 cells aimed at reconstructing normal tissue architecture and barrier function via the application of fluid flow-induced shear stress^87^. Since then, an increasing number of organ-on-a-chip devices have been reported, including advancements such as the incorporation of stretchable membranes for cell support to simulate aspects of lung function via a breathing motion^90^. These principles have been adapted to an intestinal device based on Caco-2 cells. Here cyclic peristalsis-like motions in combination with low shear stress via fluid flow resulted in the formation of a highly polarised epithelium with a buckling surface^91^. The authors of the study argue that this mimics intestinal villi architecture; however, buckling phenomena is often incurred in cell culture models, as a consequence of over-proliferation, and it remains to be shown whether the observed structures are true villi. Follow-up studies suggested that basolateral flow below the membrane facilitates continuous removal of the WNT antagonist Dickkopf-1 and that elevated WNT-mediated proliferation facilitates the epithelial folding^92^. It remains to be elucidated whether this is via a canonical or non-canonical WNT signalling pathway, given that Caco-2 cells have mutations in the APC protein that normally regulates canonical WNT signalling. Importantly, results from other groups support that fluid flow is sufficient for epithelium proliferation and folding of the epithelial layer^93,94^. Devices like these consequently provide a tool whereby it is possible, in a reductionistic manner, to study dynamic processes mimicking the consequences of flow above and below the epithelium.

Microfluidic devices have not only been used for studying established cell lines but also for primary intestinal epithelial cells. New findings suggest that primary intestinal epithelial cells cultured in these devices, at the transcriptional level, more closely resemble the in vivo epithelium than the organoids cultured in Matrigel^95,96^. Moreover, the technology can also be applied to intestinal epithelial cells derived from hPSCs, although a comparison to their in vivo counterpart is still missing^97^. The fact that the technology can be applied to human intestinal epithelial cells makes it a very attractive technology for applications addressing the effects of microbial overgrowth and intestinal inflammation^98,99^, co-cultures with anaerobic bacteria^100,101^ and viruses^102^, and for modelling, e.g. radiation exposure^103^. Moreover, based on the observation from cancer cell lines that flow affects autocrine and paracrine signalling, this also opens up opportunities to address how cells signal to each other within the epithelium.

Fluid flow both over and under the epithelial surface has also been important for the primary applications for intestines-on-chips focusing on bacterial–epithelial interactions^98–101^. Here, direct access to the apical surface allows bacteria exposure to the epithelial surface without compromising the epithelial barrier integrity. The fluid flow across the device here simulates the continuous removal of material observed in vivo and peristalsis-like mechanical motion of the epithelium promotes mixing of the luminal content, thereby reducing the risk for bacterial colonisation and overgrowth commonly seen in static culture systems. Many diseases, such as diabetes^104^ and IBD^105^ have been correlated with changes in gut microbiota composition. However, it remains unknown whether these observed correlations between specific diseases and microbial composition is driven by an underlying disease mechanism or disease-associated patient behaviour, and whether microbe composition precedes and triggers the disease. Here, the opportunity to provide more physiologically relevant culture conditions for the intestinal epithelial cells in the microfluidic devices is a clear advantage.

Despite the successes reported for organ-on-a-chip devices, they still require complex fluidic and pneumatic setups. In addition, the physiological relevance of the supporting porous membrane for cell support is questionable in terms of stiffness, thickness and adhesion opportunities, although of course this can be optimised. Attempts to circumvent these potential drawbacks of organ-on-a-chip devices include membrane- and pump-free models enabling the simultaneous analysis of up to 40 separate cell-covered tubes in parallel^106^. Although there are obvious advantages due to the simplicity of the system and the generation of fluid flow-induced shear stress over the cells, the system does not deplete secreted factors to the same degree as traditional microfluidics systems.

### Perspectives

We have come a long way from first focusing on cancer cell lines for studying the normal physiology to a phase looking at self-organising epithelium in more or less defined matrices. Currently, we can control the composition of the matrix and the biophysical properties, and we have added a dynamic setting where the culture systems can be controlled in 4D.

Although significant advances have been made in the past decade, especially with the introduction of organoid technology, there are still major challenges to overcome to develop more in vivo-like models. Presently, there is a distance between the technological and the biological developments within the field. Technological advances in biomaterials and micro-engineering fields are complemented by simpler biological studies most often using cell lines, e.g. Caco-2 cells as a first phase, whereas more advances in biological systems studying primary epithelial cells or hPSC-derived cells are performed in simpler microenvironmental systems often involving Matrigel. More advanced studies are required to address physiological questions that go beyond simple cell behaviour and represent an arena where there will be tremendous potential in combining the advances in micro-engineered devices with primary cell derivatives. A further combination of advanced topographic 3D biomaterial models with the microfluidic field is also expected.

We anticipate that the future will merge the organoid field even more with the organ-on-a-chip technology. This merge may contribute towards controlling the growth and self-renewal properties of stem cells by establishing perfusion or gradient cultures, thereby providing a more controlled biochemical microenvironment, or to provide spatial control facilitating e.g. imaging and high throughput screening. Moreover, the next step in studies of intestinal biology is to go beyond only the epithelium. Here, micro-engineered culture systems provide excellent platforms to determine the relevance in the context of defined structural confinements. This will be a first step towards generating a fully functional small intestine in vitro. With this we look forward to an exciting future and are eagerly awaiting what will come.
